# Key Factors in the Application of Sharestart to Enhance the Learning Attitude of Students

**DOI:** 10.3389/fpsyg.2021.770457

**Published:** 2021-10-20

**Authors:** Yu-Zhou Luo, Zhen Fang, Long-Jie Sun, Jie Zhu

**Affiliations:** ^1^Business School, Guilin University of Technology, Guilin, China; ^2^Administrative Office, Shanghai Jiao Tong University Affiliated Sixth People's Hospital, Shanghai, China; ^3^Faculty of Foreign Languages, Shanghai University of Medicine and Health Sciences, Shanghai, China

**Keywords:** Sharestart, teaching method, learning attitude, key factor, educational policy, educational resource

## Abstract

Under the background of globalization and the popularity of distance learning ande-learning channels provided on the Internet, teaching methods that encourage the self-directed learning of students are becoming popular. There is an increasing number of domestic teachers joining in the practice for change. The various teaching methods that make the students acquire critical thinking skills can be summarized as learning by doing, critical thinking learning, multiple assessments, team discussion teaching, and cooperative learning. With the teachers of the universities in Shanghai as the questionnaire analysis objects, a total of 360 copies of questionnaires were distributed, and 256 valid copies were retrieved, with the retrieval rate of 71%. The research results are summarized as follows. (1) The “mental adaptation and engagement of students” is the most emphasized dimension, followed by the “professional development of teachers,” “administration and parent support,” and “material and teaching strategy.” (2) The top five emphasized indicators, among 14, are the ordered cultivation of self-study and thinking habits, the development of the professional community for the collaborative lesson study of teachers, the support and cooperation of the president and the administration, adoption of heterogeneous grouping, and co-learning, discussion and cooperative learning. According to the results, it is expected to propose more definite practice directions for teachers intending to attempt such a teaching method, as well as provide some specific suggestions for the first movers of Sharestart.

## Introduction

There are various teaching methods for teachers. Being limited to time, the common teaching method among teachers is narrating and organizing points in class, forcing students to recite and memorize. More than 56% of teachers agree that such a teaching method could not assist students in independent thinking. Traditional spoon-fed teachings could easily become one-way memory. When teachers concentrate on the preparation of lectures but do not grasp the real learning absorption of students, it becomes a factor in bad learning effectiveness in students that may result in them losing interest and attention. However, in order to completely teach the curriculum content in a short period and reassure the students and parents, most front-line teachers still adopt the traditional didactic teaching. Fortunately, under the background of globalization, with the application of information technology, it became more common to provide distance learning and e-learning channels on the Internet. Berigel (2017) stated that quality must be evaluated regarding the components of distance education, context, and organization. Teaching methods to encourage the self-directed learning of the students therefore emerged, and a lot more domestic teachers are joining in the practice and change. The various teaching methods that make the students acquire critical thinking skills can be summarized as learning by doing, critical thinking learning, multiple assessments, team discussion teaching, and cooperative learning. Students could clarify the difference between personal ideas and the idea of others through thinking, analysis, criticism, opinion expression, and peer interaction, to achieve critical thinking ability. Sharestart aims to cultivate the abilities of students concerning self-study, reading, thinking, discussion, analysis, induction, expression, and writing; stresses on returning the learning power to learners; and inserts the clear spring of reform into education. Researchers, in past years, have proved that Sharestart could enhance the reading comprehension of students, and the longer times for practice greatly improve the ability that students could present (Chen et al., 2016). Moreover, it could enhance learning interests and motivation, cultivate the self-learning ability of students (Lone and Lone, 2016), enhance thinking level and problem-solving ability (Asha and Al Hawi, 2016), as well as effectively cultivate the teamwork of students, and enhance curriculum participation (Pratibha, 2017) to promote presentation expressions and oral expression abilities (Chen and Yao, 2016). Despite the rich research results in the past, it seemed to focus on the possible effects of the practice of Sharestart with various research methods, such as action research, experimental research, questionnaire survey, case study, and qualitative research, to discuss the difference before and after the practice Sharestart, while the factors in the learning effectiveness of Sharestart are seldom emphasized. Most past research on the learning effectiveness of Sharestart indicated that Sharestart could positively and effectively enhance learning effectiveness, but some research results revealed no significant difference. It revealed that Sharestart was not the absolute effective teaching method; it seemed that there were potential factors in the effectiveness of Sharestart that were neglected in past studies and seldom discussed.

As a result, the key factors in the promotion of learning attitude in Sharestart are discussed in this study, expecting to propose more definite practice directions for teachers intending to adopt the teaching method and provide some specific suggestions for the first movers of Sharestart.

## Literature Review

### Sharestart

Chen et al. (2017) explained Sharestart as teachers preparing sufficient handouts and materials for the self-study of students, designing good questions for the thinking of students, and having students learn accurate expression through constant asking. The idea was to create a teaching method that allows for the self-learning of students and trains the abilities of students to self-study, read, think, discuss, express, and write. Taggart (2018) explained it as teachers producing problem-oriented handouts for the new learning model of “cooperation and competition” among student teams, returning the stage to students and transforming teachers into hosts or guides, as well as giving the power in the learning process back to students to facilitate their learning interests, increase their various abilities, and enhance the comprehensive abilities of reading, thinking, expression, and writing. Sharestart, therefore, was the best method to train the self-expression, style, and thinking of students. Azizan et al. (2018) mentioned that to apply Sharestart, teachers had to prepare sufficient data for the students to self-study, design good questions for the thinking of the students, and constantly ask questions for students to learn accurate expression. Sharestart allowed the students to become the main characters, and curiosity and thinking were the optimal power for the learning of students.

### Sharestart Key

Kim (2018) introduced the three operation details and the key of Sharestart.

(1) Brand-new production of “problem-centered” handouts

The handouts should be started from texts and extended to extracurriculars, as well as from simple to a gradual increase in difficulty, width, and depth. The handouts should be problem-centered; a piece of data is to be provided for a question; and, the data should be divided into sections to be read in short periods and concentrating on the focus of the discussions. The handouts should be increased in depth step by step so that students could learn from shallow to deep, from easy to difficult, and from narrow to broad for larger results.

(2) Student grouping

The idea of grouping comes from the learning community, which stresses random and male-female mixed collaborative learning. Grouped cooperative learning in Sharestart is done in heterogeneous groupings, providing peer learning, mutual help, and the power of diligence, which are the key success factors in Sharestart. After grouping, the group members would get nervous together, and the classmates in other groups would carefully listen to the lesson for evaluation. The entire class is learning under such a state while getting points and being evaluated, as well as honing their skills and attention in narration and listening.

(3) Guide for teachers

With Sharestart, merely enough data are provided for students, and other three-quarters are acquired through the self-study of students by reading, thinking, expression, and writing to cultivate and enhance their basic capabilities as well as retain their learning interests.

### Factors in Learning Attitude

In addition to the intelligence factor, Lin et al. (2017) proposed four major factors in learning attitude.

(1) Psychological factors: individual motivation of achievement, personality traits, self-concept, anxiety, personal mental adaptation, attitudes, and learning habits.
(2) Physiological factors: health conditions, visual, and hearing impairments.
(3) Social factors: family background, parents' occupation, education attitude, cultural background, and community cultural value.
(4) Educational factors: teaching methods, curriculum contents, and teaching materials.

Pai et al. (2020) pointed out the intervening variables in predicting educational achievement with the background of the students; the social hierarchy of the family of the students would not directly affect the educational achievement, but the material conditions, intelligence factors, motivation of achievement, level of ambition, education attitude, cultivation styles, value and concept, language styles, and learning environment (Cenk and Deniz, 2018) could intervene.

Üzüm and Pesen (2019) proposed three factors in learning attitude.

(1) Learner factors: ability, motivation, and development level.
(2) Teacher's teaching factors: teaching hours, teaching methods, and teaching quality.
(3) Surroundings factors: family, classroom environment, and peer relationship.

Lawrence and Hanitha (2017) directly classified the factors in the learning attitudes of learners into five categories.

(1) The personal factors of the pupil: the personal psychological and physiological factors of the pupil.
(2) The family factors of the pupil: family background, family educational resource, parents' occupation, socioeconomic status, educational value, educational expectation, parent–child interaction, language type, cultural capital, and community cultural value.
(3) The teaching factor of the teachers: the teaching styles, teaching expectation, teaching methods, teaching beliefs, material content, curriculum arrangement, class management, and class culture of the teachers.
(4) School environment factor: The curriculum plan of the school, extra-school activity, teaching resources, school size, reward and punishment system, local of school, school climate.
(5) Governmental policy factor: educational policy, educational resource, environmental indicator, and result indicator.

Rani (2017) generalized the opinions of several researchers and proposed five major factors in learning attitude.

(1) Student factors: learning motivation, learning strategy, learning ability, learning style, intelligence quotient, prior knowledge, self-efficacy, learning participation, age, and gender.
(2) Teacher factors: teaching method, teaching attitude, evaluation method, and teacher–student interaction.
(3) Material factors: curriculum design, material and language, and material content.
(4) Family factors: parents' socioeconomic status, parents' education, cultivation methods, and teaching styles.
(5) Other factors: learning environment, residence area, and information integrated teaching.

Saglam and Arslan (2018) generalized the factors in learning attitude into individual factors, overall factors, and contextual factors.

(1) Individual factors: individual background factors (covering gender, cultural capital, and socioeconomic status of parents) and personality trait factors (including self-expectation, learning motivation, interests, and anxiety).
(2) Overall factor: the town where the school is located, geographic location, the school attendance of students, school size, climate, and resources.
(3) Contextual factors: referring to peer effect, teacher effect, and class culture.

## Research Design and Method

### Delphi Method

The analytic hierarchy process dimensions in this study are built according to the Delphi method. The Delphi method, also named expert survey, applies communication to send questions to various experts, inquire for opinions, and collect all the opinions of the experts to organize them into comprehensive opinions. The comprehensive opinions and predicted questions are returned to the experts for their feedback and further opinions. The experts would modify the original opinions according to the comprehensive opinions. With several times of inquiry, the consistent predicted results are acquired step by step. Such a method presents that broad representativeness and is more reliable.

### Analytic Network Process

The analytic network process, which is mainly applied to decision-making problems, is derived from Analytic Hierarchy Process (AHP) to cope with the situations in real society. Many decision-making problems cannot be expressed with the AHP structure, mainly because there are network relationships among the upper, middle, and lower hierarchies in real situations, rather than top-down linear relationships. Data from fast literature revealed that most affairs or principles related to people presented mutual dependency. For this reason, it is more appropriate to analyze with ANP than AHP in this study to better meet the practical needs.

### Establishment of Indicators

The questionnaire in this study was sent to experts in various fields through email, and an expert conference was called to develop the key factors in the enhancement of learning attitudes with Sharestart, containing the professional development of teachers, material and teaching strategy, the mental adaptation and engagement of students, and administration and parent support. Such key factors were used as the ANP dimensions, and the ANP questionnaire was built with the corresponding classifications as the criteria. The research criteria through the modification of the Delphi Method are shown below.

(1) The professional development of teachers: the development of the professional community for the collaborative lesson study of teachers, questioning guides and mastering discussion time, real-time feedback and generalization, and open classroom.
(2) Material and teaching strategy: the production of problem-oriented handouts, adoption of heterogeneous grouping, making good use of an award system, and the application of multiple assessments.
(3) The mental adaptation and engagement of students: the cultivation of self-study and thinking habits, co-learning and discussion and cooperative learning, and training the ability to publish on stage.
(4) Administration and parent support: the support and cooperation of the president and administration, encouraging teachers to participate in related studies, and parents agreeing with and supporting teaching.

### Research Subject

A total of 64 regular institutions of higher learning in Shanghai, including 39 universities and 25 colleges were selected. The Army Naval Medical University (Second Military Medical University, Shanghai, China) which started to recruit students without military status in 2018, was included in the list to have a total of 65 schools with 40 universities. For the analysis, 300 copies of the questionnaire were distributed to the teachers of the universities in Shanghai, and 256 valid copies were retrieved, with a retrieval rate of 71%.

## Data Analysis Result

After completing all hierarchical weights, the indicators were distributed according to their relative importance to show the importance of indicators in the entire evaluating system. The overall weight of the factors in learning attitude with Sharestart are generated, as shown in Table 1.

**Table 1:** The overall weight of key factors in the enhancement of learning attitude with Sharestart.

**Dimension**	**Hierarchy 2 weight**	**Hierarchy 2 order**	**Indicator**	**Overall weight**	**Overall order**
Professional development of teachers	0.258	2	Development of a professional community for the collaborative lesson study of teachers	0.112	2
			Questioning guide and mastering discussion time	0.085	6
			Real-time feedback and generalization	0.034	13
			Open classroom	0.052	10
Material and teaching strategy	0.226	4	Production of problem-oriented handouts	0.069	8
			Adoption of heterogeneous grouping	0.098	4
			Making good use of an award system	0.022	14
			Application of multiple assessments	0.038	12
Mental adaptation and engagement of students	0.277	1	Cultivation of self-study and thinking habits	0.128	1
			Co-learning and discussion and cooperative learning	0.091	5
			Training the ability of publishing on stage	0.057	9
Administration and parent support	0.239	3	Support and cooperation of president and administration	0.106	3
			Encouraging teachers to participate in related studies	0.075	7
			Parents agreeing with and supporting teaching	0.043	11

## Discussion

The premise to request for the active learning attitude of students relies on the students being able to understand with self-reading without comprehension difficulty. In this case, the provision of more comprehensible data or a large quantity of supplements is necessary. Teachers could consider teaching some content in advance and then ask the students to self-study. Most of the provided data could be studied in class and the quantity of the additional materials will depend on the difficulty and importance of the lesson, and all data are divided into small units. Students are not requested to recite, and the reading time is not long; the point will be to think. In this case, the goal of the cultivation of self-study and thinking habits are “quality” not “quantity,” “depth” not “floating,” and “cultivation of ability” not “recitation of knowledge” as well as having self-study and reading become the learning attitude of students. Meanwhile, administration departments of schools should plan time for the lesson co-preparation of teachers, with teaching teams for brainstorming and resource sharing, and give support and assistance as much as possible. Teachers are encouraged to observe lessons from other places or guidance groups of regional education units, and professional Sharestart lecturers are introduced to assist in the instruction in schools. The cooperation, observation, and reflection among teachers will result in the practical work being operated smoothly, teachers receive backing support, parents present better agreement and affirmation, and students enhance their learning attitude. Teachers, through professional learning communities and interaction and discussion with other teachers in the same fields, could cohere to the professional literacy, collaboratively make unit learning handouts (lesson preparation), and study how to operate Sharestart and precede teaching observation (lesson observation), as well as precede discussion and review after classes (lesson study). The promotion of the professional literacy of teachers aims to reinforce the active, effective, continuous, and deepen the learning attitudes of students and to return classroom focus to the learning attitudes of students. When adopting heterogeneous groupings, teachers should make complements of the difference among group members, arrange the students such that those with higher ability sit in the middle to assist their classmates with weaker levels of ability, and regularly change groups to provide the disadvantaged students with opportunities to learn from peers and excellent students for deepening the learning attitude. In this case, heterogeneous grouping does not simply encourage teamwork of the students, but also encourages competition among groups; peer stimulation and constraint could enhance the learning attitude and performance of students at different levels.

## Conclusion

According to the empirical result analysis, the following conclusions were acquired.

Among the dimensions in Hierarchy 2, the “mental adaptation and engagement of students,” weighted 0.277 and is about 27.7% of the overall weight making it the most emphasized dimension, followed by the “professional development of teachers” (weighted 0.258), “administration and parent support” (weighted 0.239), and “material and teaching strategy” (weighted 0.226). Accordingly, the mental adaptation and engagement of students is the most emphasized dimension in the enhancement of learning attitudes with Sharestart. From the overall weights of the factors in the enhancement of learning attitudes with Sharestart, the top five emphasized indicators, among 14, are the cultivation of self-study and thinking habits, development of a professional community for the collaborative lesson study of teachers, the support and cooperation of the president and administration, adoption of heterogeneous groupings, and co-learning and discussion and cooperative learning.

The research results reveal that the “mental adaptation and engagement of students,” with the highest significance, is considered as the major factor by experts. The success of Sharestart lies in the mental adaptation and engagement of students such that the cultivation of the abilities of students to self-study, co-learn, and publish on stage is important, and there should be more investments in educational resources. The effective enhancement of the mental adaptation and engagement of students could be started from “administration and parent support” and the “professional development of teachers,” with the president leading the promotion of Sharestart, the full support of the administration, and the concept of communication with parents for better comprehension, support, and agreement. Moreover, the promotion of Sharestart teachers in professional fields to receive encouragement and present passion could enhance the enthusiastic engagement of students and encourage them to acquire achievement and self-confidence from learning. What is more, multi-activation materials and teaching strategies should be adopted to enhance the automatic, interactive, and common-good learning of students.

## Data Availability Statement

The original contributions presented in the study are included in the article/supplementary material, further inquiries can be directed to the corresponding author/s.

## Ethics Statement

The present study was conducted in accordance with the recommendations of the Ethics Committee of the Guilin University of Technology, with written informed consent being obtained from all the participants. All the participants were asked to read and approve the ethical consent form before participating in the present study. The participants were also asked to follow the guidelines in the form in the research. The research protocol was approved by the Ethical Committee of the Guilin University of Technology.

## Author Contributions

Y-ZL performed the initial analyses and wrote the manuscript. ZF, L-JS, and JZ assisted in the data collection and data analysis. All authors revised and approved the submitted version of the manuscript.

## Conflict of Interest

The authors declare that the research was conducted in the absence of any commercial or financial relationships that could be construed as a potential conflict of interest.

## Publisher's Note

All claims expressed in this article are solely those of the authors and do not necessarily represent those of their affiliated organizations, or those of the publisher, the editors and the reviewers. Any product that may be evaluated in this article, or claim that may be made by its manufacturer, is not guaranteed or endorsed by the publisher.
